# Variation of Structure and Cellular Functions of Type IA Topoisomerases across the Tree of Life

**DOI:** 10.3390/cells13060553

**Published:** 2024-03-21

**Authors:** Kemin Tan, Yuk-Ching Tse-Dinh

**Affiliations:** 1 Structural Biology Center, X-ray Science Division, Advanced Photon Source, Argonne National Laboratory, 9700 S. Cass Avenue, Lemont, IL 60439, USA; 2 Department of Chemistry and Biochemistry, Florida International University, Miami, FL 33199, USA; 3 Biomolecular Sciences Institute, Florida International University, Miami, FL 33199, USA

**Keywords:** topoisomerase, type IA, supercoiling, genome topology, genomic instability, genetic diseases

## Abstract

Topoisomerases regulate the topological state of cellular genomes to prevent impediments to vital cellular processes, including replication and transcription from suboptimal supercoiling of double-stranded DNA, and to untangle topological barriers generated as replication or recombination intermediates. The subfamily of type IA topoisomerases are the only topoisomerases that can alter the interlinking of both DNA and RNA. In this article, we provide a review of the mechanisms by which four highly conserved N-terminal protein domains fold into a toroidal structure, enabling cleavage and religation of a single strand of DNA or RNA. We also explore how these conserved domains can be combined with numerous non-conserved protein sequences located in the C-terminal domains to form a diverse range of type IA topoisomerases in Archaea, Bacteria, and Eukarya. There is at least one type IA topoisomerase present in nearly every free-living organism. The variation in C-terminal domain sequences and interacting partners such as helicases enable type IA topoisomerases to conduct important cellular functions that require the passage of nucleic acids through the break of a single-strand DNA or RNA that is held by the conserved N-terminal toroidal domains. In addition, this review will exam a range of human genetic disorders that have been linked to the malfunction of type IA topoisomerase.

## 1. Introduction

The double helical structure of DNA can lead to topological barriers during cellular processes, including replication, transcription, recombination, and repair. Topoisomerases are ubiquitous enzymes that can resolve topological barriers and also regulate the level of global and local supercoiling of the DNA genome. The pursuit of enzyme activities that can alter the topological state of DNA led to the discovery of *Escherichia coli* topoisomerase I (named ω protein initially) by James C. Wang [1,2]. Purification and characterization of additional topoisomerases, including bacterial DNA gyrase [3] and mammalian topoisomerase I (called DNA untwisting enzyme initially) [4] by others showed that the different topoisomerases act by distinct mechanisms [5] for coupling DNA strand passage with cutting and rejoining of DNA to change the DNA topology and solve the various topological problems in the genome [6,7]. Sequence and structural information further demonstrated that there are different families and subfamilies of topoisomerases [5,8,9]. Members of the type IA topoisomerases subfamily share the common features of cleaving a single-strand of DNA to form the covalent complex with the 5′-phosphoryl end of the cleaved DNA [10], requiring divalent ions for catalytic activity [11,12,13]. In the strand passage model of enzyme mechanisms, the segment of DNA being cleaved is referred to as the G-segment. This cleavage of DNA enables a “gate” to open, allowing for the transportation of another DNA segment, called the T-segment, across the break. The subsequent religation of the G-segment leads to a change in DNA topology. These intermediate steps require extensive conformational changes in the topoisomerase structure to open and close the gate [11,12]. If the T-segment DNA is the complementary strand of the G-segment in a double-stranded DNA, the catalytic cycle results in a change in DNA supercoiling. If the T-segment is from another DNA molecule, a decatenation reaction can be catalyzed. Type IA topoisomerases are the only topoisomerases that have been shown to catalyze topological changes in RNA substrates [14,15]. Table 1 shows some representative examples of the distribution of various type IA topoisomerases (Topo I, Topo III, reverse gyrase) in species across Archaea, Bacteria, and Eukarya. Among these type IA topoisomerases [16], Topo I primarily functions in the relaxation of negatively supercoiled DNA to prevent excessive underwinding. In contrast, Topo III is crucial for resolving replication and recombination intermediates through its decatenation activity. Topo I and Topo III can be recognized based on the differences in their conserved amino acid sequences proximal to the active site (Figure S1). Reverse gyrase is the sole topoisomerase capable of utilizing the energy from ATP hydrolysis via its helicase domain to catalyze positive supercoiling of DNA.

Four conserved domains of type IA topoisomerases form a toroid structure first observed in *E. coli* Topo I (EcTopoI) [17,18] that contains the essential catalytic tyrosine [19] and Toprim residues for binding of divalent ions [20,21,22]. Sequence alignment (Figure S1) shows that Topo III enzymes have a strictly conserved lysine [23,24] that follows the first Toprim glutamate not found in a similar position in bacterial Topo I, and also a conserved proline that follows the catalytic tyrosine not seen in bacterial Topo I. Table I shows that Archaea and Eukarya species almost always have one or two Topo III present, while Bacteria species have at least one Topo I, but Topo III is not always present. One or two reverse gyrase with a helicase-like domain linked to the conserved type IA topoisomerase domain capable of the introduction of positive supercoiling [25,26,27] can be found in hyperthermophiles among archaeal and bacterial species [28,29]. Additional sequences that follow the type IA topoisomerase domain in Topo I and Topo III have diversified in evolution [16,30]. These sequences in the C-terminal domains of Topo I and Topo III can interact with either nucleic acid substrates or protein partners to facilitate the various type IA topoisomerase functions in cellular processes [31,32,33,34,35,36]. Recent insights into the structural organization and physiological functions of type IA topoisomerases are reviewed here.

## 2. Conserved N-Terminal Domains

### 2.1. Organization of N-Terminal Domains

**Figure 1:**
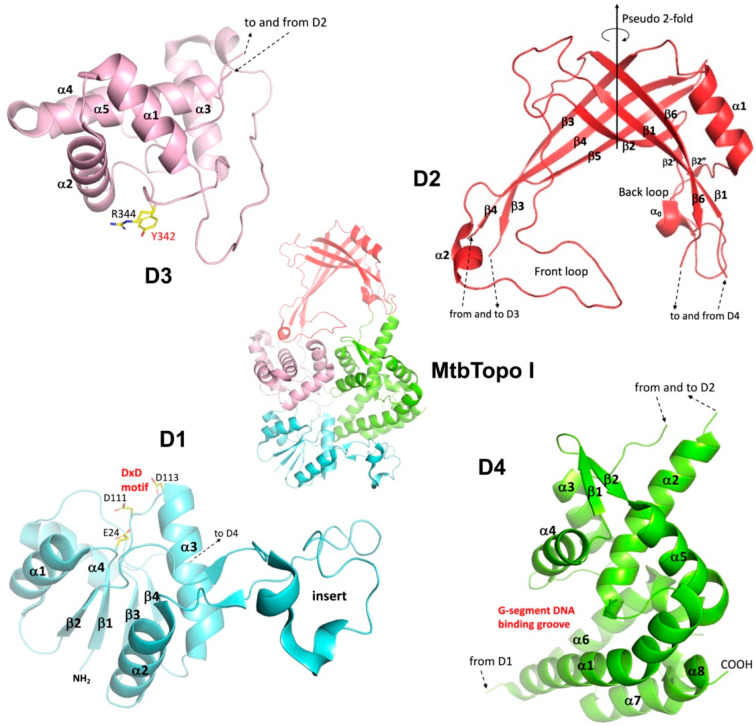
The Assembly and Features of N-terminal Domains. The four N-terminal domains D1–D4 are colored in cyan, red, pink, and green, respectively. The connections between domains are marked with arrowed dash lines. The figure is prepared based on the *Mycobacterium tuberculosis* Topo I (MtbTopo I) structure (PDB code: 5UJ1). For display purposes, the individually displayed domains are not drawn to the same scale.

Topo I and Topo III both have well-conserved N-terminal domains and variable C-terminal domains. The N-terminal region of Topo I and Topo III has a common toroidal-shaped architecture, consisting of four individual domains (D1–D4). A unique feature of four-domain toroidal assembly is that the polypeptide chain travels back and forth within these domains (Figure 1). The arrangement not only ensures connectivity between adjacent domains but also permits flexibility between them, which is crucial for the conformational changes necessary for multiple steps in the catalytic cycle of these enzymes.

D1 is a typical Toprim (topoisomerase-primase) domain, a catalytic domain found in a broad range of proteins for catalyzing the breakup and formation of a phosphodiester bond [20,37,38]. The α/β fold domain is comprised of a central four-stranded parallel β-sheet, flanked by two α helices on each side (Figure 1). The secondary structures of D1 are quite conserved in Topo I and Topo III. The absence of the β4 strand in *Streptococcus mutans* Topo I is an exception [39]. The Toprim domain hosts three conserved acidic residues that contribute to the catalytic site, one glutamate on the loop between β1 strand and α1 helix, and two aspartates in a DxD motif on the loop before α3 helix (Figure 1).

A Toprim domain often has an insert(s) for added function(s) [20]. D1 of Topo I and Topo III is characterized by a long insert between the β2 strand and α2 helix (Figure 1). The insert is mostly independent of the core of D1 in its conformation. It has a minor contribution to the DNA-binding groove within D4, the major binding site of G-segment DNA. That was the reason why this insert was assigned as a part of D4 in our earlier reports [34,40,41]. However, for the convenience of the description of domain arrangement and consistency within other studies, we will refer to this protruding insert as a part of D1.

The arch-like D2 was described earlier by Berger et al. [42] as a six-stranded anti-parallel β-barrel though it looks significantly different from a common β-barrel with two sets of extended β-strands (two hairpin-like motifs) that constitute the two sides of the structurally stable arch (Figure 1). The arch itself is rather rigid and exhibits limited expansion even when a dsDNA is trapped within the central cavity [43]. Furthermore, the order of strands of the β-barrel is the same as the ferredoxin-reductase-like β-barrel [44], which has a pseudo-2-fold symmetry and two symmetry-related extended loops. A major part of the long loop between β5 and β6 strands is the charged front central loop. The loop between β2 and β3 strands on the back of the toroidal cavity contains multiple secondary structures, including the prominent α1 helix, as well as a charged sequence motif. The two loops in the front and on the back of the toroid cavity may both play a related role in the regulation of T-segment motion in and out of the central cavity [43]. Additionally, D2 seems to resemble the oligonucleotide/oligosaccharide-binding-fold (OB-fold), which is formed by two sets of three-stranded antiparallel β-sheets. The two β-sheets are packed through hydrogen bonds between their edge strands to form a β-barrel, commonly characterized by an α-helix at one end and DNA-binding site at the other end [45,46]. Since OB-fold proteins play critical roles in the maintenance of genome integrity [45,47], it could be interesting to explore the evolution pathway of D2.

D3 is a domain of a four- or five-helix bundle. The first three and the last helices are conserved. The helical domain carries the most important catalytic tyrosine and a conserved neighboring arginine on the loop between the second and third helices (Figure 1). The helical bundle domain itself does not have any significant conformational change upon G-segment or simultaneous G- and T- segments binding to Topo I [31], even though there is a substantial movement of the domain with respect to other domains. The presence of a possible DNA-binding helix-turn-helix (HTH) motif in the domain, like α1 and α2 helices and their linker, was once discussed [42] for the possibility that it could participate in DNA binding by inserting the HTH motif into the T-segment DNA major groove, particularly when the DNA is trapped inside the toroidal cavity. However, the determination of the T-segment binding structure of *Mycobacterium tuberculosis* Topo I (MtbTopoI) rules out this possibility and supports a binding mechanism that primarily involves interactions between positively charged and polar residues present on the rim of the central cavity and the DNA backbone of one T-segment strand [43].

**Figure 2:**
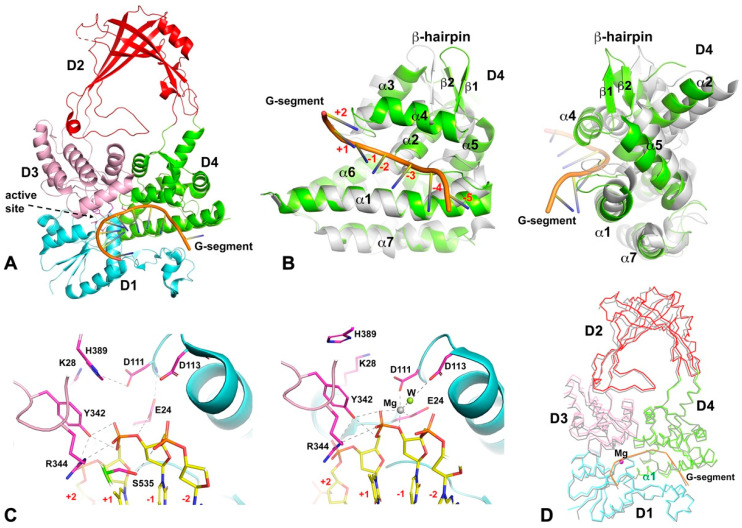
The binding of the G-segment and conformational change in the presence/absence of Mg^2+^ ions. (**A**) An example of G-segment binding with N-terminal domains of MtbTopoI. The catalytic residue Y342 is drawn in stick format to indicate its position. The G-segment ssDNA is colored in orange. (**B**) The front and side views of the conformational changes of D4 upon G-segment binding. The ribbon diagram of D4 in grey represents the structure before G-segment binding (PDB code: 5UJ1). The structure in green represents the structure after binding (PDB code: 6CQI). The nucleotide positions of the G-segment DNA at the active site are marked on the left panel. A β-hairpin motif formed by β1 and β2 strands and the turn between them is used to indicate conformational change. (**C**) The catalytic sites in the absence or presence of Mg^2+^ ion (PDB codes: 6CQI and 6CQ2) are shown in the left and right panels, respectively. The green sphere labeled as W in the right panel represents a water molecule coordinated by the Mg^2+^ ion. Hydrogen bonds are drawn using dashed lines. (**D**) The conformational change induced by the presence of Mg^2+^ ions. The structure in grey represents a G-segment bound structure in the absence of Mg^2+^ ions (PDB code: 6CQI). The structure in colors represents the G-segment binding structure in the presence of Mg^2+^ ion (PDB code: 6CQ2). The two structures were aligned based on the α1 helix of the D4 domain.

D4 has eight well-defined helices and one β-hairpin motif (Figure 1). This is the only N-terminal domain that exhibits noteworthy intra-domain conformational change, particularly upon the binding of G-segment DNA. The conformational change upon G-segment binding includes a rearrangement of helices so that the domain can tightly wrap the G-segment inside the DNA-binding groove (Figure 2A,B). The energy required for such an intra-domain conformational change is likely derived primarily from its interactions with G-segment DNA of a minimum length. Earlier experimental data indicate that a six-base ssDNA could not be cleaved by EcTopoI [48], which indicates the energy released from a six-base ssDNA’s interaction with the binding groove does not suffice to overcome the energy barrier for the intra-domain conformational change to place the oligo in a position for cleavage by the catalytic tyrosine.

### 2.2. Formation of Catalytic Site and the Presence of Mg^2+^ Ion

The key residues contributing to the catalytic site of Topo I and III are spatially separated in the apo structure (Figure 1). The conformational change induced by the binding of the G-segment ssDNA moves the conserved glutamate and DxD motif in D1, the catalytic tyrosine and a conserved arginine in D3 close to one another, thereby forming a catalytic site proximal to the sugar-phosphate backbone of the ssDNA (Figure 2C). In this configuration, the catalytic tyrosine forms two hydrogen bonds with the scissile phosphate, one to the bridging 5′-oxygen and one to a non-bridging O atom. The strictly conserved neighboring arginine binds the G-segment ssDNA with a salt bridge and a hydrogen bond to a non-bridging O atom of the scissile phosphate. Additionally, the conserved glutamate from D1 participates in the formation of a hydrogen bond to the bridging 3′-oxygen of the scissile phosphate. Interestingly, the two aspartates from the DxD motif do not interact with the G-segment directly. Based on the conformation of the active site, a G-segment cleavage mechanism that involves proton relay was proposed [22]. A covalent bond is subsequently formed between the catalytic tyrosine and the 5′-end of the cleaved G-segment, which creates a covalent intermediate.

### 2.3. Interdomain Movements Observed from Crystal Structures

As discussed earlier, D4 is the only N-terminal domain that undergoes intra-domain conformational change upon G-segment binding (Figure 2B). Alternations in the relative domain–domain orientation and inter-domain movements serve as the mechanistic foundation for the conformational changes adopted by four-domain N-terminal toroidal assembly during the distinct steps of the topoisomerase catalytic cycle (Figure 2D). The first evidence of interdomain movement was observed in the crystal structure of an EcTopoI fragment containing D2 and D3 only [49]. This structure shows the interdomain flexibility with a large relative rotation (up to 52°) between these two domains and, importantly, suggests a possible mechanism of opening the N-terminal toroid assembly for the transfer of the T-segment. However, such a large inter-domain rotation has not yet been observed in a full-length N-terminal domain structure. The Apo form of N-terminal domains is considered to be in a closed conformation, in which D1 and D4 associate with D3 through a range of non-covalent interactions to maintain the conserved toroidal shape. In a G-segment bound pre-cleavage or post-religation state [22,50], D3 moved away from D1 to create an interdomain space and an extended binding site for the 3′-end of the G-segment. The interdomain movement also reduces the contact between D3 and D4. Interestingly, in a crystal structure with a cleaved G-segment and the formation of a covalent intermediate [40], it is noteworthy that despite a cleaved single-stranded oligonucleotide substrate covalently attached to its catalytic tyrosine, D3 does not swing away from D1 and D4 to allow for the opening of the gate between D1 and D3. It seemingly suggests that interactions with a short oligo substrate used in crystallization may not provide sufficient driving force to facilitate the opening of the gate.

Molecular packing inside the crystal may restrict the observation of all interdomain movement and related conformation of the enzyme throughout the various stages of its catalytic cycle. However, crystal structure can still provide insights into certain domain–domain movements relevant to the conformational change required for its functions. Our recently published MtbTopoI structure in complex with both G- and T-segments may represent a catalytic stage right after G-segment religation and before T-segment release form the central cavity [43]. The new structure provides more detailed information on inter-domain movement in comparison to Apo and G-segment bound-only structures. For example, the 3′ regions of the G-segment and D1 move closer to each other, creating a second kink of the G-segment. This association might potentially serve as a preparatory step for D3 to move away from D1 to open the protein gate. The movement of D3, in a combination of translation and rotation, reduces its contact with D1 and D4 significantly [43].

The arch-like D2 is expected to exhibit a certain level of flexibility in its connection to D3 and D4 because of its two loop-like links to each of these two domains. Small back-and-forth movement of D2 has been observed in apo structures from different crystals of EcTopoI [18,51] and MtbTopoI [22,41]. The relative D2 movement observed in G- and/or T-segment bound structures [31,34,40,43] could be a result of conformational changes induced by DNA-binding and molecular packing combined. However, in the structures with only G-segment bound, D2 tends to rotate backward slightly [22,40,50].

The two hinge joints from D2 to D3 and D4 have not been well defined. The core of the arch-like D2 is rigid, while its two extended side bases could serve as hinge joints (or parts of hinge joints) for the relative rotation of D2 with respect to D3 and D4. When the T-segment is captured inside the central bound structure, the distance between the two side bases at the bottom of D2 expands by about 3 Å [43]. In the early EcTopoI D2-D3 domains structure [49], the relative rotation between these two domains was attributed to being through a “break point”, which is a part of the D2 side base that connects to D3. Further exploration is needed to understand how this D2 movement is related to the mechanism of gate opening-and-closing.

## 3. Variable C-Terminal Domains

### 3.1. C-Terminal Structural Motifs

**Figure 3:**
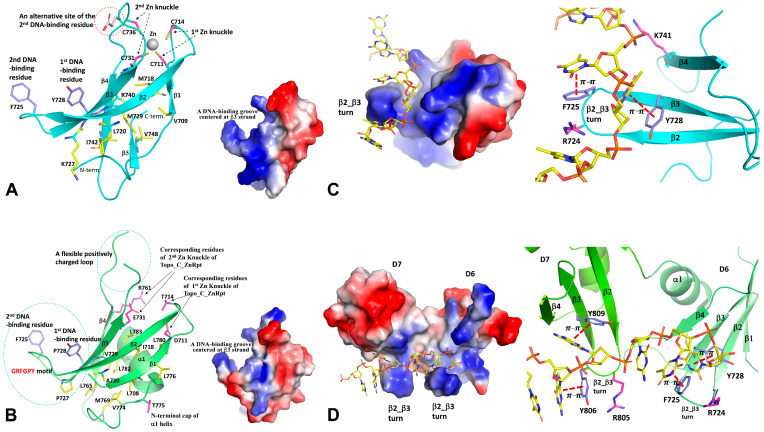
The structures and DNA-binding properties of two Types of C-terminal domains, Topo_C_ZnRpt and Topo_C_Rpt. (**A**) The structure of a typical Topo_C_ZnRpt domain, represented by *E. coli* Topo I (EcTopoI) D7 domain. The Zn knuckle forming cysteines (in magenta), the hydrophobic core residues (in yellow), and the key DNA binding residues (in blue or grey) are drawn in stick format and labeled. On the right side of the figure is an electrostatic potential surface representation of the D7 domain. The figure shows a blue groove (with negative electrostatic potential) for potential DNA binding. (**B**) The structure of a typical Topo_C_Rpt domain, represented by *M. smegmatis* Topo I (MsmTopoI) D6 domain. The residues corresponding to the Zn knuckle-forming residues in the Topo_C_ZnRpt domain (Figure 3A) are drawn in stick format (in magenta) and labeled for comparison. The capping residue at the N-terminus of the α1 helix is also drawn in magenta to highlight its position. The hydrophobic core residues and the key DNA binding residues are displayed in yellow and blue or grey sticks, respectively. The break in the β3_β4 loop demonstrates the structural flexibility of the loop. On the right side of the figure is an electrostatic potential surface representation of the MsmTopoI D6 domain, which shows a similar blue groove for potential DNA binding as that of the EcTopoI D7 domain shown in Figure 3A above. The MsmTopoI D7 domain, which is not drawn in this figure, has similar DNA-binding features. (**C**) The interaction between EcTopoI D7 and ssDNA. The left side figure is an overview of ssDNA binding within the blue DNA-binding groove of EcTopoI D7. The right-side figure shows the molecular details of the interaction. (**D**) The simultaneous interaction of two MsmTopoI C-terminal domains, D6 and D7, with ssDNA. The left side figure is an overview of ssDNA binding within the extended DNA-binding groove formed by MsmTopoI D6 and D7 domains. The right side figure shows the molecular details of the interaction.

In contrast to highly conserved N-terminal domains, the C-terminal domains of Topo I and III are characterized by the variation in their sequences, as well as their numbers and orders of structural motifs. Two initially identified C-terminal domains are Topo_C_ZnRpt and Topo_C_Rpt, which are the two major types of C-terminal domains in bacterial Topo I [30]. The Topo_C_ZnRpt type domain contains a C4 zinc finger, which is relatively easy to recognize from the amino acid sequence, particularly in cases when there are multiple repeats of these domains. The five C-terminal domains of EcTopoI represent the prototype of the Topo_C_ZnRpt domain [52,53]. From the first to the last, they also show the degeneration of the C4 zinc finger during evolution and/or gene duplication. A typical Topo_C_ZnRpt domain is a four-stranded antiparallel β-sheet locked by a Zn-binding site formed by four cysteines on the top of the domain (Figure 3A) [34]. The first identifiable CXXC motif is situated immediately after the first strand, creating a so-called knuckle on β1_ β2 loop (first Zn knuckle, Figure 3A). The other two ligand cysteines are on the β3_ β4 loop, forming a second but non-canonical knuckle (second Zn knuckle, Figure 3A) with a variable spacer between two cysteines. According to the classification of Zn-fingers [54], the 4-Cys Zn finger fold formed from two knuckles with each from a β-hairpin is called a zinc ribbon [53,55]. Here, we consider the β1 strand, β1_β2 loop, and β2 strand together as one hairpin, and the β3 strand, β3_β4 loop, and β4 strand together as the second hairpin. The two hairpins make an angle because of the twist of the β-sheet. Therefore, domains D5, D6, and D7 of EcoTopo1 are called zinc ribbon domains [53]. Apparently, the 4-Cys zinc ribbon fold at one end of the domain helps stabilize these small β folds. The last two C-terminal domains (D8, D9) of EcTopoI retain the zinc ribbon fold [53,56] but have lost the cysteines for Zn-binding. The Topo_C_ZnRpt, along with other types of zinc finger motifs, such as zf-GRF and zf-CCHC, are present in Topo III sequences across the domains of life [30,33,57]. The zf-GRF in *Caenorhabditis elegans* Topo III has been shown to be important for its function in recombination [58]. While zinc fingers are absent in the C-terminal domains of Topo III from *Saccharomyces cerevisiae* and other *Ascomycota* fungi, they are present in Topo III from many other fungal species [30].

The Topo_C_Rpt domain does not contain any zinc finger-forming cysteine residues. It was identified based on the repeats of sequence motifs in the C-terminal region of Topo I in *Actinobacteria*, and its existence was first confirmed in MtbTopoI [41]. The four Topo_C_Rpt domains present in *M. tuberculosis* and *M. smegmatis* Topo I C-terminal domains (D5-D8) are considered prototypical, as they are each composed of an antiparallel four-stranded β-sheet and a C-terminal helix packed on one side of the twisted β-sheet (Figure 3B). The signature motif, G(R/K)(Y/F)GPY, is located at the β2_β3 turn and its vicinity. The highly positively charged insertion in the D6 domain of some Topo_C_Rpt domains forms part of the β3_β4 loop, which is partially disordered due to its high flexibility (Figure 3B). Interestingly, the crossing-over helix (α1) is capped by a very conserved threonine residue, which (residue number i) forms two hydrogen bonds using its sidechain to the amide group of i+3 and i+4 residues, respectively. The interaction between the α1 helix and β-sheet is highly hydrophobic, and their interface area forms a major part of the domain core. The α1 helix apparently stabilizes the four-stranded β-sheet of the Topo_C_Rpt domain.

### 3.2. Interaction between C-Terminal Domains and Nucleic Acid Substrates

Both Topo_C_ZnRpt and Topo_C_Rpt are characterized by a common four-stranded β-sheet [30]. A highly conserved aromatic residue (first DNA binding site) is situated in the middle of the third strand, β3, which plays an important role in the interaction with ssDNA (Figure 3A,B) [31,34]. The interaction primarily involves π–π stacking between the sidechain of the aromatic residue and the nucleotide base of ssDNA (Figure 3C,D). These domains also contain a second nucleotide-binding site, which is contributed by another aromatic residue (Figure 3A,B). In Topo_C_ZnRpt, the aromatic residue of the second binding site could be located on the turn between β2 and β3 strands (β2_β3 turn) or on the loop between β3 and β4 strands (Figure 3A) [34]. A sequence motif G(R/K)(Y/F)G on the β2_β3 turn usually indicates a second nucleotide-binding site (Figure 3C). In Topo_C_Rpt, there is a common R(Y/F) motif on the β2_β3 turn (Figure 3B) [31]. The aromatic Y/F residue serves as the second nucleotide binding site through π–π stacking (Figure 3D) in a similar manner to the first binding site. Interestingly, the arginine, in turn, does not interact directly with DNA substrate in the currently available crystal structures (Figure 3C,D) [31]. It is possible that arginine may be involved in the initial substrate recruitment. Generally, one Topo_C_ZnRpt or one Topo_C_Rpt domain can bind two consecutive nucleotides of a ssDNA substrate (Figure 3C,D). It is likely that any domains lacking characteristic aromatic residues may not participate in direct interaction with the ssDNA substrate.

Despite limited biochemical and structural information available for the zf-GRF and zf-CCHC C-terminal domains present in eukaryotic Topo III, the known functions of these small zinc-finger-containing homology domains found in other proteins [59,60], as well as structural modeling [30], suggest that they are potential ssDNA binding domains, similar to Topo_C_ZnRpt and Topo_C_Rpt, and may also function as RNA binding domains [61,62].

In addition to these repeated structural motifs, the C-terminal domains of type IA topoisomerases can also utilize other sequence elements to interact with nucleic acid substrates. One instance of such elements is the long lysine repeats that confer high enzyme processivity for Topo I from *Streptomyces* and other *Actinobacteria* [32,63]. The C-terminal arginine–glycine–glycine (RGG) box present in human TOP3B and other related Topo III [57] has also been shown to be required for both RNA and DNA topoisomerase activity [64].

## 4. Physiological Functions of Type IA Topoisomerases

### 4.1. Importance of Protein–Protein Interaction Partners

Protein partners that interact directly with type IA topoisomerases can modulate the physiological functions of individual topoisomerase by either enhancing the activity and stability of the topoisomerase protein or directing the topoisomerase to a specific cellular location where the type IA topoisomerase activity is needed. In *E. coli*, direct interaction between Topo I and RNA polymerase is important for the relaxation of transcription-driven negative supercoiling and prevention of R-loop accumulation by Topo I [36,65,66,67], while *E. coli* Topo III has been shown to interact physically and functionally with the DnaX complex at the replication fork to remove precatenanes [68]. Human TOP3A forms a dissolvasome complex with RMI1, RMI2, and BLM helicase for DNA repair [69,70,71,72], while human TOP3B is stabilized in a complex with TDRD3 [64,73,74] for regulation of mRNA translation and turnover [75] as well as promotion of transcriptional activation and repression in response to starvation [76]. Lastly, the direct interaction between reverse gyrase and various DNA repair proteins in Archaea is likely to correlate with its role in genome stability [29].

### 4.2. Resolution of Replication, Recombination, and Repair Intermediates in Bacteria and Archaea

It has been suggested that at least one type IA topoisomerase can be found in the genome of nearly every organism because of the ability of type IA topoisomerases to resolve topological entanglements encountered in replication, recombination, or repair that require cutting and rejoining of a single strand of DNA [77]. The requirement for overcoming these topological barriers can probably be satisfied largely by the presence of the structurally conserved toroidal N-terminal domains that form the active site for DNA cutting-rejoining. Topo III, encoded by the *topA* gene in archaea *Sulfolobus solfataricus*, has been shown to act as a decatenase [78]. Deletion of *topA* in *Sulfolobus islandicus* resulted in slow growth and defects in cell cycle control consistent with the involvement of Topo III decatenation activity for chromosomal segregation [79]. *E. coli* Topo III is much more efficient in DNA decatenation than Topo I due to shorter pauses between decatenation cycles [80]. The relatively greater efficiency of decatenation activity over relaxation activity for Topo III is likely to be conserved for Topo III encoded by *topA* in Archaea. *E. coli* Topo III can unlink daughter chromosomes during DNA replication along with Topo IV [81]. Topo III activity partially rescues Topo IV deficiencies probably by removing precatenanes within single-strand regions near the replication fork [68,82].

### 4.3. DNA Supercoiling, Transcription and Regulation of R-Loops by Bacterial Topo I

The relaxation of negatively supercoiled DNA by bacterial Topo I countering the supercoiling activity of gyrase plays an important role in the homeostatic regulation of global DNA supercoiling in bacteria, as shown first in *E. coli* [83,84,85,86] and more recently for other bacteria [87,88,89]. The crystal structure of full-length EcTopo I, with ssDNA bound to the C-terminal domains [34], suggests that the C-terminal Topo_C_ZnRpt domains (or zinc ribbon domains) strongly and specifically interact with the ssDNA in the negatively supercoiled region of DNA. This interaction may be the root of the efficient recognition and suppression of hypernegative supercoiling of DNA. The interactions observed between the Topo_C_Rpt domains of MsmTopoI and ssDNA [31] may play a similar role in the efficient relaxation of negatively supercoiled DNA by bacterial Topo I with the C-terminal Topo_C_Rpt domains.

DNA supercoiling is highly dependent on transcription, as predicted by the model of Liu and Wang [90], with Topo I relaxing the negative supercoils in the DNA template behind the advancing polymerase and gyrase, relaxing the positive supercoils ahead of the polymerase. The direct protein–protein association of the C-terminal domains of Topo I with RNA polymerase has been demonstrated in biochemical studies for both *E. coli* and *M. smegmatis* [36,67,91]. More recent genome-wide ChIP-Seq analysis showed colocalization of *M. tuberculosis*, *Streptococcus pneumoniae*, and *E. coli* Topo I with RNA polymerase, often at promoters and during transcription elongation [66,92,93]. Interaction between transcribing RNA polymerase and Topo I C-terminal domains is required to prevent R-loop formation in *E. coli* [66]. Topo I deficiency leads to constitutive stable DNA replication (cSDR) from R-loop [94] and excessive transcription–replication conflicts that result in lethality [95]. There have been conflicting reports for a long time on whether *E. coli topA* null mutants are viable without compensatory mutations. Although compensatory mutations appear frequently in gyrase genes [83,84], *E. coli topA* null mutants can also acquire mutations rapidly in other genes that reduce cSDR and transcription–replication conflicts [94,95,96,97]. Amplification of *parC* and *parE* genes coding for Topo IV has often been observed to compensate for the loss of bacterial Topo I relaxation activity in *topA* null mutants [98,99,100]. Overproduction of Topo IV can also allow the isolation of viable *topA topB* null mutants of *E. coli* and *B. subtilis* by preventing over-replication from R-loops [96,101,102].

When Topo I and gyrase are the only type IA and type IIA topoisomerases present in a bacterial species, Topo I is expected to be essential. In these bacteria, Topo III is not available to provide overlapping activities, and there are no Topo IV genes for providing relaxation activity [103] or amplification [96,99] that would rescue the *topA* null mutants. The essentiality of Topo I has been demonstrated for *M. tuberculosis* and *M. smegmatis* that do not have Topo III or Topo IV [104,105,106]. Results from saturation mutagenesis also indicated that Topo I is essential for *Mycobacterium abscessus* [107]. The obligate intracellular bacterium *Chlamydia trachomatis* has Topo I and gyrase as the only topoisomerases. Knockdown of *topA* transcription by CRISPRi downregulated expression of late developmental genes and impaired the *C. trachomatis* developmental cycle [89]. *Helicobacter pylori* has no Topo III or Topo IV but has multiple *topA* genes coding for Topo I (Table 1). Transposon mutagenesis suggested that for at least one of these Topo I (corresponding to A0A402E4A0 in Table 1), the C-terminal region with four zinc fingers cannot be disrupted without causing lethality [35].

Although *Pseudomonas aeruginosa* has Topo IV, failure to obtain a knockout mutant of Topo I (corresponding to A0A431XC87 in Table 1) suggested that this Topo I is essential [108]. The mutant that lacked the last 59 amino acids at the C-terminal region was shown to be viable but showed pleiotropic effects on the type III secretion system (T3SS), phenazine production, antibiotic susceptibility, and biofilm formation [108]. *Streptococcus pneumoniae* has both Topo III and Topo IV. Nevertheless, Topo I has been reported to be essential for the growth of *S. pneumoniae*, and the ratio of GyrA:Topo I determines the level of supercoiling [88]. Overexpression of either wild-type *S. pneumoniae* Topo I or a mutant Topo I that has reduced relaxation activity affects global transcription differentially [109].

### 4.4. Function of Reverse Gyrase

The roles of topoisomerases in DNA topology regulation in Archaea should differ from Bacteria based on the topoisomerases present [29]. Gyrase activity is not always present in Archaea [110], and no type IA Topo I with robust relaxation activity has been identified in Archaea. The type IIB Topo VI present in archaeal species is capable of relaxing both positive and negative supercoils, in addition to possessing decatenase activity [29,111]. The histone variants [112] and nucleoid-associated proteins (NAP) present can also constrain and modulate DNA supercoiling for regulation of DNA topology in Archaea [113,114] similar to NAPs in Bacteria [113,115,116], with mechanisms that may include direct stimulation of topoisomerase activities [117,118,119,120]. In the hyperthermophilic archaeon *S. solfataricus*, it was reported that homeostatic control of DNA supercoiling is mainly mediated by the fine-tuning of TopR1, one of the two reverse gyrases [121]. Genetic studies suggested that the other reverse gyrase TopR2 in *Sulfolobales* may have another more important function [122]. Based on previous work, the requirement of reverse gyrase activity for hyperthermophiles [28,123] may be related to DNA protection and repair in addition to positive DNA supercoiling [29,124,125].

### 4.5. Function of TOP3A in Eukaryotes

Targeted inactivation of mouse TOP3A showed that this type IA topoisomerase is essential for early embryonic development [126]. In the course of DNA repair, efficient displacement loop (D-loop) disruption during homologous recombination by BLM helicase is facilitated by the association of BLM with the TOP3A–RMI1–RMI2 complex [69,127]. Exonuclease DNA2-mediated resection of broken DNA ends as the first step of homologous recombination, is also stimulated by the TOP3A–RMI1–RMI2 complex together with the MRE11–RAD50–NBS1 (MRN) complex [127]. Targeting *Arabidopsis thaliana* TOP3A activity to Holliday junction-like DNA repair intermediates requires the C-terminal zinc finger domain [33]. Mutations in human TOP3A result in a Bloom syndrome-like disorder with increased sister chromatid exchanges (SCEs) [128]. It was found that in ATRX wild-type pediatric osteosarcomas with alternative lengthening of telomeres (ALT), TOP3A is required for proper BLM localization, and TOP3A amplification promotes ALT DNA synthesis [129]. Recent large-scale genome-wide association studies (GWAS) and functional studies identified TOP3A as one of the ovarian endometriosis risk-associated genes, possibly due to the role of DNA repair and homologous recombination in endometriosis [130].

TOP3A can be localized both in the nucleus and mitochondria [131,132]. Mitochondrial TOP3A has been shown to be required for the maintenance of Drosophila mtDNA genome integrity [132,133], decatenation, and segregation of human mtDNA [134]. The type IA human TOP3A in mitochondria contributes to mtDNA replication and mtDNA topology control [135,136] along with the type IB TOP1MT [7]. It has been suggested that impaired mitochondrial metabolism plays an important role in the pathogenesis of TOP3A-deficient Bloom syndrome-like disorder [137,138]. A homozygous variant in the TOP3A gene has been associated with multiple mtDNA deletions [137]. Chronic progressive external ophthalmoplegia (CPEO) plus syndrome due to pathogenic biallelic missense variants in the TOP3A gene may be associated with reduced TOP3A activity, affecting its mitochondrial functions [134,139]. Based on the correlation of the disease characteristics and positions of the different pathological TOP3A variants on the crystal structure of TOP3A N-terminal domains, it has been proposed that variants with more severe effects on TOP3A catalytic activity could lead to Bloom syndrome-like disorder, while variants expected to have less severe impact on TOP3A catalytic activity could result in adult-onset mitochondrial disease [140].

### 4.6. Function of TOP3B in Eukaryotes

The function of TOP3B could be based on its interaction with either DNA or RNA as a substrate, potentially affecting both transcription in the nucleus and translation in the cytoplasm. The role of TOP3B–TDRD3 in neurodevelopment and disorders such as schizophrenia and cognitive impairment was first discovered [64,141] in association with Fragile X Mental Retardation 1 protein (FMRP), an RNA-binding protein essential for synaptic formation [142]. TOP3B deficiency in mice has later been shown to induce changes in neuro-behaviors and alterations in connectivity between the different brain regions [143]. Characterization of TOP3B or TDRD3 knockout mice suggested defective neuronal activity-dependent transcription as well as defective post-transcriptional regulation as mechanisms behind the cognitive impairment [144,145]. A study of TOP3B or TDRD3 knockout in human HCT116 colon cancer cells indicated that TOP3B–TDRD3 can regulate mRNA translation and turnover with mechanisms that may not always depend on the TOP3B topoisomerase activity [75]. Additional analysis of these mutant HCT116 cells demonstrated disrupted transcription for both starvation-activated genes and starvation-repressed genes in response to starvation [76]. A study in Drosophila suggested that TOP3B may act as an RNA topoisomerase in siRNA-guided heterochromatin formation and transcriptional silencing [146].

Methyl arginines in the TOP3B RGG box near its C-terminus mediate interactions between FMRP and TOP3B-TDRD3 [64,147]. Substitution of TOP3B methyl arginines with lysines resulted in reduced in vitro activity for relaxation of negatively supercoiled DNA, accumulation of transcription-associated R-loops in vitro and in cells, as well as reduced stress granule localization for TOP3B [147]. Helicase DHX9 has been identified as an interaction partner of the TDRD3/TOP3B complex to suppress promoter-associated R-loops [148]. TOP3B also forms a complex with the helicase DDX5 independent of TDRD3 for the resolution of R-loops [149]. TOP3B can suppress R-loops by more than one mechanism [149]. In addition to the relaxation of hypernegatively supercoiled DNA behind the RNA polymerase complex, TOP3B can decatenate the intertwined DNA and RNA strands after unwinding the R-loop by cutting either the DNA or RNA strand to create a gate for passage of the other strand of the R-loop [149]. Increased R-loop from loss of TOP3B function in a patient with bilateral renal cancer has been linked to genome instability [150].

### 4.7. Cellular RNA Substrates for RNA Topoisomerase Activity

Human TOP3B has been shown to associate with mRNAs and form a covalent cleavage complex [151], indicating that certain mRNAs are cellular substrates for their RNA topoisomerase activity. mRNAs bound by TOP3B include those encoding proteins with neuronal functions related to schizophrenia and autism [64]. Results from eCLIP-seq analysis of HCT116 cells showed that TOP3B preferentially binds coding regions of long mRNAs [75]. Analysis of in vitro cleavage site preference on RNA did not reveal any sequence preference for cleavage by human TOP3B [24]. It is possible that RNA structure or interacting partners of TOP3B may direct TOP3B to specific RNA substrates. The TOP3B-TDRD3 complex has been reported to be required for efficient replication of positive-sense ssRNA viruses [152]. However, it was recently shown that TOP3B is dispensable for replication of murine coronavirus [153]. While TOP3B knockout may have an effect on ZIKA virus replication [152], the latest result suggested that TOP3B does not have a universal role in promoting the replication of positive-sense ssRNA virus.

Formation of covalent catalytic intermediate between bacterial or archaeal type IA topoisomerases and cellular RNA has not been demonstrated even though they can also act as RNA topoisomerases in biochemical assays in vitro [14,15]. While TOP3B from animals have TDRD3-dependent association with polyribosomes, no polyribosome association could be detected for type IA topoisomerases from yeast and *E. coli* for possible function in translation [14]. Based on the accumulation of rRNA precursors in an *M. smegmatis* Topo I knockdown strain and the rescue of rRNA processing deficiency in RNase E knockdown cells by Topo I expression, it was proposed that mycobacteria Topo I participates in rRNA processing [154].

## 5. Conclusions

The universal presence of type IA topoisomerases and their catalytic activity on RNA substrates suggest that the ancestor of this subfamily of topoisomerases may act on RNA genomes in early life forms. The exact physiological functions of type IA topoisomerases currently found in Archaea remain to be fully elucidated. The fusions and gene duplications of unique domains to the catalytic core of type IA topoisomerases allow the individual topoisomerases to adapt to their specific roles in cellular physiology. A closer examination of the sequence and domain organization of type IA topoisomerases that have not been studied in detail could offer additional examples of the spectrum of potential functional roles for this important class of genome regulators.

The crystal structures of type IA topoisomerases have provided static pictures of the domain organizations and binding interactions with DNA substrates. Transient intermediates with large conformational changes have been proposed in the model for catalysis. Single-molecule studies have been useful for providing support for the mechanistic model [155,156], but a structure of the critical intermediates with the toroid in a fully open conformation has not been observed directly. Future studies with cryo-EM could provide more information on conformations that are obstructed in crystal structures due to crystal packing or other limitations.

There has been increased interest in type IA topoisomerases since the report of the RNA topoisomerase activity of TOP3B and the associated function in neurological development [64,141]. While the ability to utilize RNA as substrate in vitro is conserved in other type IA topoisomerases throughout evolution [14], it remains uncertain if their RNA topoisomerase activities have significant physiological functions. It is also unclear how the interactions with DNA and RNA substrates may differ. Replacement of the DNA substrates in available crystal structures with RNA substrates would require some rearrangements of type IA topoisomerase residues. It would be very helpful for future studies to utilize separation-of-function mutations or small molecule probes that selectively target the enzyme activity on either DNA or RNA substrate alone without affecting the other. Additionally, new three-dimensional structures of type IA topoisomerases with RNA substrates bound would provide valuable insights into the mechanism of substrate selectivity and interaction with RNA.

## Figures and Tables

**Table 1:** Examples of type IA topoisomerases present in organisms across different domains of life. UniProt Knowledgebase (UniProtKB) Accession Numbers are shown in red for sequences that have C-terminal zinc finger motifs, in green for sequences that have C-terminal Topo_C_Rpt motif, and in purple for sequences that have both zinc finger and Topo_C_Rpt motifs. Sequences that have other potential C-terminal nucleic acid binding motifs are shown in blue.

Species	Protein	Gene(s)	Length (# AA)	UniProtKB Accession Number
Archaea				
*Sulfolobus solfataricus*	Reverse gyrase 1	*rgy1*	1,242	Q97ZZ8
*Sulfolobus solfataricus*	Reverse gyrase 2	*rgy2*	1,166	Q97ZF5
*Sulfolobus solfataricus*	Topo III	*topA*	668	Q97ZJ8
*Nanoarchaeum equitans*	Reverse gyrase	*NEQ434*, *NEQ318*	575, 701	Q74M79,Q74MA4
*Nanoarchaeum equitans*	Topo III	*NEQ045*, *NEQ324*	408, 172	Q74N66,Q74M99
*Methanosarcina mazei*	Topo III	*MSMAW_1096*	832	A0A0E3PWI6
*Methanosarcina mazei*	Topo III	*MSMAW_1564*	752	A0A0E3PXP2
*Thermoplasma volcanium*	Topo III	*topA*	770	Q97CT2
*Nitrososphaera viennensis*	Topo III	*topA*	697	A0A060HRZ8
Nitrosopumilus maritimus *	?	*Nmar_1311*, *Nmar_1632*	124, 152	A9A2G8, A9A650
*Thermoplasmatales archaeon*	Topo I	*BRD56_01955*	1067	A0A2P6VYX4
*Thermoplasmatales archaeon*	Topo I	*BRD56_11190*	732	A0A2P6VTI6
Bacteria				
*Escherichia coli*	Topo I	*topA*	865	P06612
*Escherichia coli*	Topo III	*topB*	653	P14294
*Thermotoga maritima*	Reverse gyrase	rgy	1104	O51934
*Thermotoga maritima*	Topo I	*topA*	633	P46799
*Mycobacterium tuberculosis*	Topo I	*topA*	934	P9WG49
*Deinococcus radiodurans*	Topo I	*topA*	1021	Q9RUL0
*Agrobacterium tumefaciens*	Topo I	*topA*	892	A9CJ93
*Caulobacter crescentus*	Topo I	*topA*	899	Q9A5J6
*Chlamydia trachomatis*	Topo I	*topA*	857	A0A0H3MCD9
*Helicobacter pylori*	Topo I	*topA_4*	746	A0A402E4A0
*Helicobacter pylori*	Topo I	*topA_3*	686	A0A402E2T6
*Helicobacter pylori*	Topo I	*topA_5*	644	A0A402E598
*Neisseria gonorrhoeae*	Topo I	*topA*	768	A0A7H9WPJ8
*Neisseria gonorrhoeae*	Topo III	*topB_1*	679	Q5EP76
*Neisseria gonorrhoeae*	Topo III	*topB_2*	753	A0A7H9WG49
Eukarya				
*Saccharomyces cerevisiae*	Topo III	TOP3	656	P13099
*Ustilago maydis*	Topo III	UMAG_11929	986	A0A0D1C790
*Choanephora cucurbitarum*	Topo IIIα	TOP3A	749	A0A1C7N0U0
*Choanephora cucurbitarum*	Topo IIIβ	TOP3B	548, 290	A0A1C7NLX2, A0A1C7NMP8
*Trypanosoma brucei brucei*	Topo IIIα	Tb11.01.1280	918	Q383X7
*Trypanosoma brucei brucei*	Topo IIIβ	Tb11.01.0910	841	Q384B1
*Trypanosoma brucei brucei*	Topo IAmt	Tb10.70.5940	806	Q38C52
*Giardia intestinalis*	Topo IIIα	DHA2_154636	896	V6TB71
*Giardia intestinalis*	Topo IIIβ	*DHA2_15190*	973	V6TB61
*Arabidopsis thaliana*	Topo IIIα	TOP3A	926	Q9LVP1
*Arabidopsis thaliana*	Topo IIIβ	TOP3B	865	F4ISQ7
*Arabidopsis thaliana*	Topo IAmt	AT4G31210	1284	F4JRX3
*Homo sapiens*	Topo IIIα	TOP3A	1001	Q13472
*Homo sapiens*	Topo IIIβ	TOP3B	862	O95985

* It is not clear if *Nitrosopumilus maritimus* has a functional type IA topoisomerase.

## Data Availability

No new data were created.

## Supplementary Materials

The following are available online at https://www.mdpi.com/article/10.3390/cells13060553/s1, Figure S1: Alignment of type IA topoisomerase active site region residues.
