# Supplementary material for: Variation of Structure and Cellular Functions of Type IA Topoisomerases across the Tree of Life
Source: Cells. 2024 Mar 21;13(6):553. doi: 10.3390/cells13060553 (PMC10969213; doi:10.3390/cells13060553)
Supplement: Supplementary file 1 [file cells-13-00553-s001.zip › cells-2898231-supplementary.pdf]

Topo III

Sulfolobus solfataricus	Q97ZJ8	11	LIIAEKSKAAKK	314	GLISYPRTNS
Nanoarchaeum equitans	Q74N66	2	IIIAEKPSVAKR	289	GYISYPRTSS
Methanosarcina mazei	A0A0E3PWI6	4	VAFAEKNKAAAQ	313	GFTSYPRTET
Methanosarcina mazei	A0A0E3PXP2	3	LIVTEKNIAARR	313	GYISYPRTDN
Thermoplasma volcanium	Q97CT2	7	VIIAEKVDAGRR	309	GLISYPRTDN
Haloferax volcanii	A0A384LCM2	7	LIITEKDNAARR	322	GYITYPRTDN
Escherichia coli	P14294	3	LFIAEKPSLARA	324	KLITYPRSDC
Neisseria gonorrhoeae	Q5EP76	3	LFLCEKPSQAKD	318	KITSYPRTPC
Neisseria gonorrhoeae	A0A7H9WG49	5	LIIAEKPDMAKS	297	KVLTYPRSDF
Saccharomyces cerevisiae	P13099	4	LCVAEKNSIAKA	352	GFISYPRTET
Ustilago maydis	A0A0D1C790	4	LCVAEKPSIAKS	342	GFLSYPRTET
Choanephora cucurbitarum	A0A1C7N0U0	4	LCVAEKPSAAKK	327	GLISYPRTET
Choanephora cucurbitarum	A0A1C7NLX2	7	LMVAEKPSLAES	333	GYISYPRTET
Trypanosoma brucei brucei	Q383X7	5	LNVAEKPSVARE	320	GYISYPRTET
Trypanosoma brucei brucei	Q384B1	5	LMVAEKPSIAES	322	GYISYPRTES
Giardia intestinalis	V6TB71	6	LCVTEKNSVAAE	405	GLISYPRTET
Giardia intestinalis	V6TB61	3	LLIAEKPSIAEM	324	GYISYPRTES
Arabidopsis thaliana	Q9LVP1	12	LNVAEKPSVAKS	338	GFISYPRTET
Arabidopsis thaliana	F4ISQ7	8	LMVAEKPSIALS	327	GFISYPRTES
Homo sapiens	Q13472	37	LCVAEKNDAAKG	358	GYISYPRTET
Homo sapiens	O95985	5	LMVAEKPSLAQS	332	GYISYPRTET

Topo I

Thermoplasmatales archaeon	A0A2P6VYX4	6	LVIVESPAKVDT	321	GHITYMRTDS
Thermoplasmatales archaeon	A0A2P6VTI6	4	VIVVESGAKTRT	300	ALITYMRTDS
Thermotoga maritima	P46799	8	YIVVESPAKAKT	284	AFITYMRTDS
Mycobacterium tuberculosis	P9WG49	20	LVIVESPTKARK	338	GYITYMRTDS
Deinococcus radiodurans	Q9RUL0	48	LVIVESPAKAKT	365	GYITYMRTDS
Agrobacterium tumefaciens	A9CJ93	3	VVVVESPAKAKT	299	GLITYMRTDG
Caulobacter crescentus	Q9A5J6	3	VVVVESPAKAKT	298	GLITYMRTDG
Neisseria gonorrhoeae	A0A7H9WPJ8	5	LLIVESPSKAKT	300	GLITYMRTDS
Helicobacter pylori	A0A402E4A0	4	LIIVESPAKAKT	293	GVITYMRTDS
Helicobacter pylori	A0A402E2T6	5	VIIIESPNKVAK	290	GLITYIRTDA
Helicobacter pylori	A0A402E598	6	VFIIENPNKIAK	297	GLITYIRTDS
Pseudomonas aeruginosa	A0A431XC87	5	LVIVESPAKAKT	320	GYITYMRTDS
Pseudomonas aeruginosa	A0A431X2A2	3	LVIIEAPGKLKK	294	GLITYHRTDN

Reverse gyrase

Sulfolobus solfataricus	Q97ZZ8	621	LFIVESPNKAKT	961	GLITYHRTDS
Sulfolobus solfataricus	Q97ZF5	583	LLIVESPTKAKT	899	GLITYHRTDS
Nanoarchaeum equitans	Q74MA4	43	LFIVESPNKART	348	GLITYHRTDS
Methanopyrus kandleri	Q49600	781	LMIVESPNKARM		
	Q8TYK7			74	GLITYHRTDS
Pyrococcus abyssi	Q9UZ86	641	LMIVESPNKART	951	GLCTYHRTDS
Conexivisphaera calida	A0A4P2VAE5	658	LLLVESPTKART	986	GLITYHRTDS
Thermotoga maritima	O51934	544	LIIVESPTKAET	847	GFITYHRTDS

Mitochondrial Topo IA

Trypanosoma brucei brucei	Q38C52	12	LVIVESPNKVIK	358	GHITYPRTDS
Arabidopsis thaliana	F4JRX3	476	VIVVESMTKAKI	775	GLITYMRTDG

Figure S1. Alignment of type IA topoisomerase active site region residues. Resides around the first strictly conserved TOPRIM glutamate and the catalytic tyrosine for formation of the covalent intermediate are shown here. Archaeal sequences are highlighted in yellow. Residues conserved in all type IA topoisomerases are shown in red. A lysine that always follows the conserved TOPRIM glutamate in topo III sequences is shown in green.
